# Care pathways for people with major depressive disorder: A European Brain Council Value of Treatment study

**DOI:** 10.1192/j.eurpsy.2022.28

**Published:** 2022-06-15

**Authors:** Rebecca Strawbridge, Paul McCrone, Andrea Ulrichsen, Roland Zahn, Jonas Eberhard, Danuta Wasserman, Paolo Brambilla, Giandomenico Schiena, Ulrich Hegerl, Judit Balazs, Jose Caldas de Almeida, Ana Antunes, Spyridon Baltzis, Vladmir Carli, Vinciane Quoidbach, Patrice Boyer, Allan H. Young

**Affiliations:** 1 Department of Psychological Medicine, Institute of Psychiatry, Psychology & Neuroscience, King’s College London, London, United Kingdom; 2 Centre for Mental Health, University of Greenwich, London, United Kingdom; 3 Division of Psychiatry, Department of Clinical Sciences, Lund University, Lund, Sweden; 4 National Centre for Suicide Research and Prevention of Mental Ill-Health, Karolinska Institutet, Stockholm, Sweden; 5 Department of Neurosciences and Mental Health, Fondazione IRCCS Ca’ Granda Ospedale Maggiore Policlinico, Milan, Italy; 6 Department of Pathophysiology and Transplantation, University of Milan, Milan, Italy; 7 Department of Psychiatry, Psychosomatics and Psychotherapy, Goethe University, Frankfurt, Germany; 8 Institute of Psychology, Eötvös Loránd University, Budapest, Hungary; 9 Department of Psychology, Bjørknes University College, Oslo, Norway; 10 Chronic Diseases Research Center, Nova Medical School, Nova University of Lisbon, Lisbon, Portugal; 11 European Brain Council, Brussels, Belgium

**Keywords:** Care pathways, diagnosis, major depressive disorder, treatment

## Abstract

**Background:**

Despite well-established guidelines for managing major depressive disorder, its extensive disability burden persists. This Value of Treatment mission from the European Brain Council aimed to elucidate the nature and extent of “gaps” between best-practice and current-practice care, specifically to:

1. Identify current treatment gaps along the care pathway and determine the extent of these gaps in comparison with the stepped-care model and
2. Recommend policies intending to better meet patient needs (i.e., minimize treatment gaps).

**Methods:**

After agreement upon a set of relevant treatment gaps, data pertaining to each gap were gathered and synthesized from several sources across six European countries. Subsequently, a modified Delphi approach was undertaken to attain consensus among an expert panel on proposed recommendations for minimizing treatment gaps.

**Results:**

Four recommendations were made to increase the depression diagnosis rate (from ~50% episodes), aiming to both increase the number of patients seeking help, and the likelihood of a practitioner to correctly detect depression. These should reduce time to treatment (from ~1 to ~8 years after illness onset) and increase rates of treatment; nine further recommendations aimed to increase rates of treatment (from ~25 to ~50% of patients currently treated), mainly focused on targeting the best treatment to each patient. To improve follow-up after treatment initiation (from ~30 to ~65% followed up within 3 months), seven recommendations focused on increasing continuity of care. For those not responding, 10 recommendations focused on ensuring access to more specialist care (currently at rates of ~5–25% of patients).

**Conclusions:**

The treatment gaps in depression care are substantial and concerning, from the proportion of people not entering care pathways to those stagnating in primary care with impairing and persistent illness. A wide range of recommendations can be made to enhance care throughout the pathway.

## Introduction

Major depressive disorder (MDD) affects over 300 million people at any time and is considered to have the largest disability burden of all illnesses [1]; treatment-resistant depression (TRD) is associated with a considerable proportion of costs [2, 3]. This enormous illness burden of MDD is known to be attributable to several factors including:

1. High prevalence, which exceeds 30 million people in Europe [4] and mood disorders overall having a 14% lifetime prevalence [5].
2. Disabling impacts of episodes and symptoms themselves, which incur severe disability across the domains of psychosocial functioning and quality of life [6]. These frequently persist despite treatment and are equivalent or greater than for many other common and chronic health conditions [7].
3. Low rates of treatment response, with early-stage TRD affecting ~50% of people receiving first-line treatments and ~30% developing into substantive TRD [8]. As well as elongating episodes, evidence indicates that people with TRD have higher rates of comorbidities, suicide risk, service use, and functional disability [9]. Critically, people with existing TRD have a reduced likelihood of future treatment responses [10], and notably a longer duration of untreated depression also represents a risk to developing TRD [11].
4. High rates of major depressive episode relapse/recurrence: although half of people who experience an MDD episode recover without relapsing, this means that 50% experience an either largely unremitting 15% course of depressive illness or a recurrent (with intermittent recovery; 35%) course [12].

**Figure 1.**
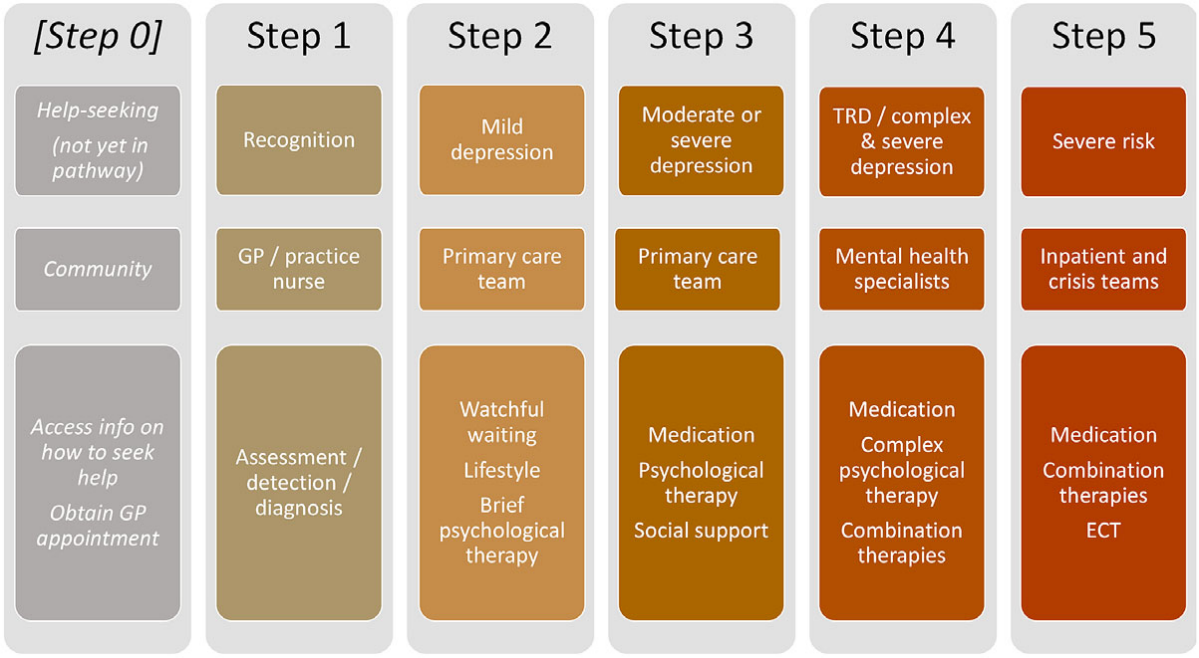
Schematic of stepped care pathway for major depressive disorder. Depiction of stepped care model for recognizing and managing depression. This reflects the stepped care model presented in the NICE depression guideline (2009). Adaptations from the original are only in the following respects: (a) level of detail (minimized here for clarity), (b) addition of a “Step 0,” which we have developed in this project as the preceding stage to entering the stepped care pathway itself, and (c) in structure of presentation, as the NICE guideline does not explicitly show the setting(s) that each step takes place in. Here, the top row displays the title/summary of that step, the middle row shows the setting within which it should be managed, and the third summarizes details of management guidelines for each step. Note that Step 5 is not considered in the current treatment gaps as this is reserved for a minority of urgent or complex cases, often following the failure of previous treatment steps.

These disabling and widespread impacts of depressive episodes (and the recurrence of episodes) persist despite numerous established treatment options with extensive evidence bases [13–15] and well-reputed guidelines for MDD management [16–18]. Guidelines typically integrate a stepped care model for MDD treatment: this is a framework for care provision where all individuals with suspected depression enter the care pathway, receiving assessment and support followed by active monitoring; subsequent steps delineate a sequential process of increasing treatment intensity based on prior response and illness severity/urgency. To the best of our knowledge, the most commonly used stepped care pathway is from the UK’s National Institute for Health and Care Excellence (NICE) depression guidelines [18]; a simplified depiction of this is shown in Figure 1 (adapted in structure and quantity, but not nature of information included). Evidence suggests that adhering to stepped care improves outcomes for those with moderate or severe depression [19]. However, the research to date has methodological drawbacks, and implementing best-practice care is challenged by resource-constrained healthcare services [20].

The 2022 Lancet–World Psychiatric Association Commission [21] brought together a range of scholars in our field and provided a broad review outlining the burden, nature, epidemiology, and treatment of depression. This culminated in recommendations in a call to “united action.” While these recommendations included appeals that would improve care pathways (e.g., early help-seeking, evidence-based treatment, and collaborative care), the article focused on the benefits of doing so (i.e., early intervention improving longer-term outcomes) rather than the current gaps between current- and best-practice care pathways, and how these could specifically be optimized. The extent to which best-practice guidelines are followed remains unestablished. Delineating precisely what happens on patients’ journeys during the full course of MDD illness (from onset onward) would allow us to determine the discrepancies between optimal and current practices. Most critically, this would allow informed and specific recommendations to improve MDD care.

### Aims and objectives

The overarching aim of the “value of treatment” (VoT) study is to develop evidence-based policy recommendations to improve the care pathway(s) for MDD in Europe. The current study was particularly focused on working age adults with MDD. To maximize the literature that could be included, MDD is defined categorically as a binary construct. We highlight that stepped care models differ based on dimensions (e.g., severity) within the group of people who meet criteria for MDD and that this dimensional approach is important in conceptualizing mental health conditions [21].

To meet the project aim, three stages were undertaken with the following objectives:

1. Identification of potential treatment gaps and patient needs along the care pathway (working group consensus);
2. Synthesis of extent and nature of each treatment gap from data gathered across six countries spanning Europe (care pathway analysis). Both of these are important steps to inform the third aim; and
3. Propose potential solutions to, in future, minimize the size of current treatment gaps identified (consensus-based recommendations).

This approach intended to provide an international representation of current and best practices in the detection and management of depression. The project aligns with the VoT’s objectives to model impacts along treatment pathways, derived from current knowledge, and incorporating expert opinion from clinicians, researchers, and patients in making recommendations to improve care. The economic costs of both the extant treatment gaps, and the implications for savings through optimizing care pathways, are clearly critical to this aim, and the economic aspects of this project are described elsewhere [22].

## Methodology

### Treatment gap identification

A non-systematic review was initially undertaken. PubMed and handsearches using key words related to depression and treatment were undertaken to identify articles that:

1. were one of the following designs: national/international MDD management guidelines, systematic reviews (with or without meta-analyses), and controlled and observational studies;
2. focused on best-practice care for depression, including diagnosis and treatment (although specific treatment strategies were not a focus); and
3. recent (published within the last decade) or highly cited (>50 citations per year) as a proxy for relevance to identifying the most pertinent treatment gaps.

The evidence review was enhanced by drawing on the network of expert collaborators (clinicians and researchers) within the VoT project and on known unpublished data. The evidence review was also used as an opportunity for scoping, to determine the extent to which the optimal MDD care pathway can be modeled, and which countries could be included in the study. The findings from these were presented to the working group, who subsequently met to reach consensus on the primary treatment gaps to focus on. Consensus was reached via unstructured discussion in a single meeting following the preceding activities.

### Synthesis of treatment gap data

A survey was first circulated to the working group, to determine which treatment gaps data could be gathered for each country (see Supplementary Material S1). The survey consisted of an iteration of each treatment gap and definition of what data could be included (as follows), with options for each working group member to select whether they had identified data for their country to provide for each treatment gap. Relevant data were then gathered and synthesized from published and unpublished reviews, observational and interventional studies, extant databases, health records, and national survey data and statistics. The summaries were prepared by summarizing the methodology and results of each, adding methodological limitations where notable, although a formal quality assessment was not undertaken and studies were not subject to prioritization based on their methodology. We report instead on all data/studies that were provided which contained information pertaining to each treatment gap, for each country.

### Recommendations to minimize treatment gaps

Since there is no clear consensus on the priorities for optimizing care pathways for MDD, the Delphi method provides a systematic approach to determine expert consensus where uncertainty cannot be resolved using experimental or epidemiological methods [23]. We employed a modified Delphi approach as described recently [24]. The stages for this study comprised the following:

1. *Recommendation item-set development:* Two experts in the field (AHY and RZ) and a facilitator (RS) developed a first set of potential recommendations to improve care pathways, using the latest substantive NICE treatment guidelines [18] in combination with the current treatment gaps identified. The items were reviewed and discussed during an initial online meeting, where challenging issues were considered in drafting the initial item set. Feedback and discussion following the initial meeting was incorporated into a final draft of the items to be circulated to the expert group.
2. *First survey circulation/completion:* The wider group of expert clinicians, researchers, and patients rated each item as to how important and relevant they considered it as a putative recommendation for optimizing the MDD care pathway. They were also provided with questions and prompts to facilitate the suggestion of additional items to be added in subsequent survey rounds. Following completion, using prespecified criteria, the results were synthesized, and based on this, each item was either accepted for inclusion (>80% agreement of the recommendation as essential or important), disregarded (<60% agreement), or reconsidered in subsequent survey (60–80% agreement).
3. *Second survey circulation/completion:* The above stage was repeated, with the items in this round consisting of those requiring reconsideration from the previous round, in addition to any new aspects that arose from the comments and suggestions in the first round. The results were synthesized as previously.
4. *Final survey circulation/completion:* The above stage was repeated, permitting respondents to propose topics and considerations for discussion in the subsequent stage.
5. *Final consensus meeting:* The group met and discussed (a) the items accepted for inclusion in the set of consensus recommendations, (b) the items rated as disregarded, (c) the items remaining with 60–80% agreement in each survey round, and (d) all comments made during the surveys and arising from prior discussions. Following agreement in this meeting, the final set of recommendations was finalized.

The six countries opportunistically focused on are Sweden, Germany, Italy, the UK, Portugal, and Hungary.

## Results

### Treatment gap identification

The agreed treatment gaps reflected care for MDD spanning the (stepped) care pathway as recommended by best-practice guidelines (see Figure 1). The reviewed evidence is incorporated into the “Synthesis of treatment gap data” section. The treatment gaps are as follows:

1. *Rates of depression detection:* Missed diagnosis prevents individuals from entering a stepped care pathway and is a well-known challenge impacting upon subsequent treatment effectiveness. Reasons for non-detection include those on the part of patients (e.g., nondisclosure of symptoms), service factors (e.g., attempting to seek help but barriers to care access), or clinician factors (e.g., inaccurate diagnosis). We note here that by clinicians, we primarily refer to general physicians, although note that other healthcare professionals can also be involved in detecting depression and therefore our consideration of “detection” is not limited to medical doctors.
2. *Time to diagnosis/treatment:* Missed diagnosis is closely linked with delays to both detection and treatment initiation for depression (and in some cases a delay to treatment after detection). After recognition/diagnosis (Step 1), treatments span the whole stepped care pathway (Steps 2–5).
3. *Rates of pharmacological/psychological treatment:* As the mainstay interventions for MDD, it was considered important to include both pharmacological (antidepressants; Steps 3–5) and psychological therapies (Steps 2–5), which have more or less [25–27] equivalent efficacy.
4. *Rates and frequency of follow-up contacts after treatment:* A key tenet of stepped-care approaches is ensuring continuity of care [28], and thus healthcare contacts are critical for monitoring treatment effects (including efficacy, tolerability, and adherence).

The above treatment gaps are considered primarily for primary care (stepped care stages 1–3; see Figure 1).

1. *Access to secondary (or psychiatric) care services:* For people whose depression cannot be adequately managed in primary care (approximately one third of patients, e.g., TRD), clearly more specialist intervention is required as in other medical disciplines.

Originally, access to specialist/tertiary services was included, since treatment managed by clinicians specializing in affective disorders is evidenced to improve outcomes for people not responding to psychiatric intervention [29], but there was an absence of available data.

### Synthesis of treatment gap data

**Figure 2.**
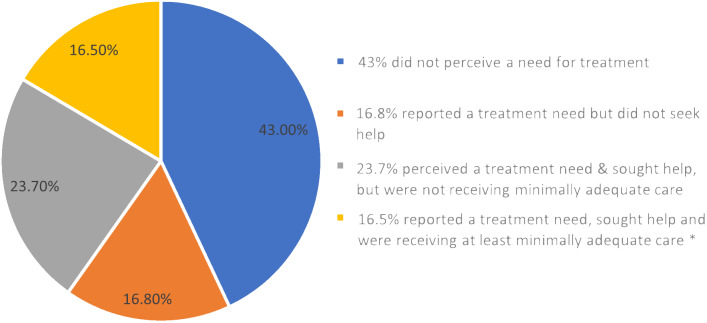
WHO world mental health survey estimates of detection and treatment rates for people with major depressive disorder (MDD). Summary of status (with regard to help-seeking and treatment receipt) of those meeting criteria for depression in the World Health Organisation (WHO) international surveys. Data were gathered from a representative set of community households across 21 countries over the decade prior to publication in 2017. The 12-month prevalence of MDD was 4.6% (adults). This shows that only 16.5% of people with MDD received “minimally adequate care.” *“minimally adequate care” is defined as at least 1 month receiving pharmacological treatment including more than four medical contacts, or more than eight sessions of psychotherapy.

The most recent WHO world mental health surveys provide indications for both treatment gaps 1 and 3. Data were gathered from a representative set of community households across 21 countries over the decade prior to publication in 2017 [30]. The 12-month prevalence of MDD was 4.6% of adults, of whom 57% reported a perceived need for treatment. For the other 43% of individuals, it is unclear if their symptoms were subclinical (despite ostensibly meeting MDD diagnostic criteria) or the lack of treatment need was perceived due to factors known to influence help-seeking, for example, stigma-related [31]. Of the patients with a perceived need for treatment, a little over two thirds of people sought help (defined as ≥1 visit to a service provider, defined more broadly than formal healthcare services). It is worth noting that this percentage might be higher if the definition of *help-seeking* included unsuccessful attempts to attend a visit to a service provider. However, only around 41% (16.5% of the whole MDD population) received minimally adequate care (defined as ≥1-month pharmacotherapeutic treatment with ≥4 medical contacts, or ≥8 psychotherapy sessions). Figure 2 displays a summary of these data described above for all individuals categorized as having MDD, that is, the proportion of respondents from the WHO surveys in terms of receipt of treatment.

Supplementary Material S2 contains a more detailed description of the data synthesized for all treatment gaps, summarized below and in Table 1.

**Table 1.** Summary of treatment gap data by country.

Treatment gap	Country	Summary of findings
1. Detection rates	International	Estimates ranging from 25 to 70% detection with most estimates between 45 and 65%.
	UK	Estimates ranging from 35 to 64%, averaging approximately 50% detection.
	Germany	Estimates ranging from 21 to 75%, averaging approximately 55% detection.
	Portugal	Uncertain/high percentage detection (single study).
	Sweden	Wide ranging estimates averaging approximately 57% detection.
	Italy	Estimates averaging detection between 30 and 64%.
	Hungary	As few as 7% of true depression cases could be detected in primary care (single study).
2. Delays to detection and treatment	International	Estimates ranging from 1 to 8 years (mode 8 years) overall depressive illness.
	UK	Average 8 years (single study), >2 years (within episode), 43% TRD patients.^a^
	Germany	Wide variation averaging ~2 years, >3 months (within episode), 66% patients (single study).
	Italy	Average 3.25 years, >6 months (within episode), 64% patients.
3. Treatment rates (pharmacological and psychological)	International	20–52% of diagnosed untreated (~70% untreated in samples including undiagnosed). Only ~25% psychological.
	UK	21–32% of diagnosed untreated (~70% untreated in samples including undiagnosed). ~25% psychological.
	Portugal	45% untreated. Most pharmacological, 38% lifetime psychological therapy (single study).
	Sweden	53% of diagnosed patients not offered treatment (actual treatment rate lower; single study).
	Italy	61–79% of diagnosed untreated (antidepressants). ~37% of diagnosed some psychological (single study).
	Hungary	55–60% diagnosed but untreated with antidepressants.
4. Follow-up rates after treatment	International	1/3 no FU within 3 months, 1/3 some FU, 1/3 adequate FU (≥3 visits within 3 months).
	UK	2/3 FU within 2 months; proportion *offered* but not *attended* FU may be slightly higher.
	Portugal	General: Average 2 and 3 primary care visits per year in treated people with MDD (single study).
	Italy	General: 60% of MDD patients had ≥1 primary care visit per month (nonspecific to MDD; single study).
5. Access to secondary care	International	~24–38% of diagnosed patients referred to secondary care (note: referral rather than contact rates).
	UK	Most estimates 5–21% (up to 44% in one unrepresentative sample of people with TRD).
	Germany	~12% of diagnosed MDD patients (single study).
	Portugal	22–28% treated in secondary services within 12 months.
	Italy	~1–10% of people with MDD in psychiatric care, although highly accessible after inpatient discharge.

*Note:* No data available from individual countries not shown.

Abbreviations: FU, follow-up; MDD, major depressive disorder.

a Additional data not reported here (not comparable in scope to most other studies).

#### Rates of depression detection

*International:* Data from WHO mental health surveys indicate a rate of diagnosable MDD detection between 40 and 70% [30]. Other international studies have estimated as low as 25% MDD detection [32], while meta-analyses have reported general practitioner (GP) correctly detecting depression in 47% of cases [33] or with sensitivity between 46 and 64% [34].

*Portugal:* The rates of accurate MDD prevalence are unknown, although national surveys indicated a 12-month MDD prevalence between 6.8 and 10% [35, 36], and the equivalent diagnosed “depressive symptoms” prevalence was 9.3% (almost a decade after the aforementioned national survey) [37].

*Sweden:* Estimates of diagnosed MDD have ranged from 1 to 8.5%, averaging around 4%, while the true MDD point prevalence may be around 7% [38–41]. Speculatively, the detection rate may be ~57% of true MDD cases.

*The UK:* National survey data indicated that just under half of people with depression/anxiety had received a diagnosis [42]; another study reported this rate to be 39% [43], and for MDD specifically, a primary care study found GPs diagnosed MDD with a sensitivity of 30–33% [44].

*Germany:* One primary care study reported that only 21% depressed individuals had an MDD diagnosis [45]. A higher diagnosis rate of up to 75% may be indicated, from other published estimates of 12-month MDD diagnosis (5.6%) [46] and surveys including undiagnosed cases (7.4%) [47].

*Italy:* Primary care studies indicated a diagnostic sensitivity of 64% [34, 48], but other estimates highlight a lack of help-seeking (between 47 and 80% of people with depressive symptoms [49, 50], so the true rate of detection depression might be as low as one third of people meeting MDD criteria.

*Hungary:* Limited data indicated that GPs diagnose MDD with a sensitivity of 6.7% [33].

#### Time to diagnosis/treatment

*International:* Survey data have suggested a median time to diagnosis of 4 years [32], or 8 years [51, 52] after onset. Other data from patients with diagnosed mood disorders indicate a median time to first treatment as low as 1 year [53].

*The UK:* One study reported an average of 8 years between mental illness onset and first healthcare contact in people with depression/anxiety [54], while within-episode (in a TRD cohort) indicated that almost half had been in episode for over 2 years before beginning antidepressant treatment [55].

*Germany:* One survey study suggested that just over one third received treatment within 3 months of episode onset, while one quarter waited for over 3 years [56].

*Italy:* One study reported an average of 3.25 years with untreated MDD [57].

#### Treatment rates

*International:* Studies of diagnosed patients have reported that (cross-sectionally), the proportion of patients not receiving treatment is approximately one third [32, 59], or as low as 15% [60]. In cohorts containing undiagnosed individuals, rates of 71% untreated [61], or 84% inadequately treated [30] have been reported. Rates of psychological therapies are consistently lower than antidepressant medications [60, 61].

*Portugal:* National survey data estimated 45% of individuals with 12-month MDD to be untreated, but rates of psychological therapy (defined flexibly) were equivalent to those treated with medication 38% [35, 36].

*Sweden:* One study reported that 47% of people with diagnosed depression were *offered* appropriate treatment [38].

*The UK:* In studies containing undiagnosed patients, untreated depression rates of 62–77% have been reported [42, 44, 62]. In studies of diagnosed patients, this rate reduces to 21–32% [43, 63]. Rates of pharmacological treatment appear at approximately twice the rate of psychological [42].

*Italy:* Primary care studies have reported that only 21–39% of MDD patients considered likely to benefit from antidepressants received them [58, 64] and 29% were untreated overall [58]. The latter study also estimated that only 12.9% of untreated patients considered likely to benefit from antidepressants started a new drug treatment [58].

*Germany:* One primary care report found that only 4% of MDD-diagnosed patients and 12% depressed patients with a different diagnosis had not received (lifetime) treatment [45].

*Hungary:* It has been reported that 55–60% of patients with a depression diagnosis are untreated [65, 66].

#### Follow-up after treatment initiation

*International:* USA studies have reported an average of one visit per month after initiating antidepressants [67, 68], 26–31% having adequate follow-up [67, 69], but 33% without follow-up by 3 months [69].

*Portugal:* National survey data indicated an average of two to four primary appointments per year for treated patients [35, 36].

*Italy:* One primary care study reported 60% of depressed patients attended more than one appointment per month [48].

*The UK:* National statistics suggest that 64% have a primary care review within 2 months of diagnosis [70], while medical records studies have reported 78% (follow-up offered) [71] and 67% (follow-up attended) [63] within 1 month of treatment initiation.

#### Access to secondary care

*International:* USA studies of people with depressive symptoms have reported 23 and 24% receiving treatment or referred to a psychiatrist/specialist [61, 72]; rates were similar 26% in diagnosed patients in a Canadian study [32] and a Dutch study [73].

*Portugal:* National survey data estimated rates of 22–28% of people with MDD referred or receiving treatment from a psychiatrist or psychologist within 12 months [35, 36].

*Italy:* A speculative estimate of 8% MDD patients receiving secondary care treatment has been reported [58, 74].

*The UK:* One study of MDD-diagnosed participants identified a referral rate of 21% to secondary care over 3 years, [43], although rates as low as 5 and 6% [63, 75] have been reported. One study of people with TRD identified a rate of 44%, but acknowledged a likely overestimation [55].

*Germany:* One study reported that 12% of diagnosed MDD patients had been referred to a specialist [45].

**Figure 3.**
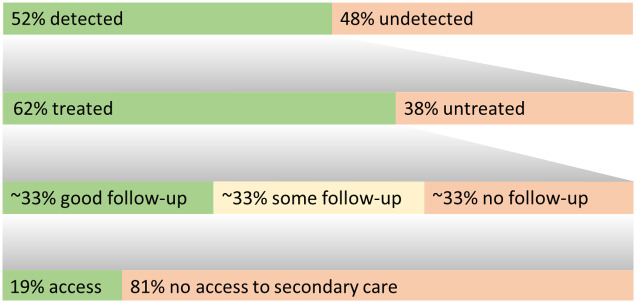
Summary graphic integrating treatment gap estimates. For treatment gaps 1, 3, 4, and 5, this graphic summarizes the estimated proportion of individuals with each outcome. The top row represents all individuals with a major depressive episode, the second represents those with a diagnosis, and the third/fourth of those treated for depression.

A simple information graphic of summary rates from treatment gaps 1, 3, 4, and 5 is presented in Figure 3.

### Recommendations to minimize treatment gaps

The expert panel was comprised of one or two mood disorders psychiatrists from each included country, plus two non-clinician research psychologists, two primary care practitioners, and three service users with MDD.

The initial survey included 35 items (see Supplementary Material S3). Amalgamating the ratings from the first round resulted in 11 items accepted for inclusion, 3 items disregarded and 21 items to be reconsidered in the second round. The second round comprised 28 items (including suggestions from the first round); after completion, a further 7 items were accepted, 7 disregarded, and 14 reconsidered. In the final survey round, 11 items were accepted and 3 unresolved. After discussion in the consensus meeting, one further item was accepted and the other two were amalgamated into existing recommendations (see Table 2).

**Table 2.** Summary of recommendations to improve care pathways for people with depression.

Treatment gap addressed		Summary of recommendation
Enhance detection/pathway entry	1a	Improved information provision to patients and those around them—how to seek help and what to expect. Should be multidomain (e.g., Internet, in care settings, other public domain, workplace, and education).
	1b	Increased service availability (GP appointment number and flexibility in terms of timing and format).
	1c	Increased duration of appointments, to maximize likelihood of depression screening. Also applies to follow-up meetings to ensure ascertainment of tolerability, adherence, and effectiveness of treatments.
	1d	Integrate self-management e-mental health tools with healthcare practice.
Improve treatment provision	2a	Development and evidence support for computerized decision-support tools to support treatment selection in line with guidelines.
	2b	Tools for robust information provision to patients about benefits and harms of different treatments. Ideally verbal discussion, otherwise, for example, (e)leaflet or in self-management tool. Must be clear, detailed, and evidence-based.
	2c	Practical information provision about psychological therapy options (different types, waiting lists, and costs).
	2d	Although 2b and 2c can inform patient preference, clinicians to encourage and *enable* treatment selection as per patient preference (requires additional resources for expanding treatment options).
	2e	Removal of barriers for patients to access more time intensive treatments where indicated (e.g., legal and cultural facilitation for time out of work/education).
	2f	Prescribing support tools, integrated with electronic health records to increase efficiency, and accurate detection of, for example, contraindications to treatment.
	2g	Increased provision of different psychological therapies (where appropriately evidenced)—ensuring appropriate trained therapists, appropriate dose/duration, cost, and provision of transition after completion.
	2h	Shared-care arrangements, for example, psychiatrists design procedures for nursing/pharmacy staff to follow with patients to manage treatment initiation (e.g., suitability, prescription, and titration).
	2i	Adding mental health workers (e.g., nurses) to primary care, for wider support to physicians, for example, psychoeducation and side effects monitoring.
Continuity of care/follow-up after treatment	3a	Optimize self-management tools, to support patients in managing their condition, for example, monitoring symptoms, side effects, and adherence, and/or accessing psychoeducation and advice.
	3b	Utilization of e-tools for healthcare practitioners to monitor patients’ self-rated symptoms/side effects and indicate need for increased or reduced follow-up appointments after treatment initiation.
	3c	Standardized assessment of symptoms and side effects by clinicians to better monitor response and tolerability (measurement-based care), with this encouraged but not mandated as a target-based exercise.
	3d	Screen for risk factors to indicate if more (or less) follow-up needed, for example, polypharmacy, history of recurrent or treatment-resistant depression, risk for bipolar or suicidality, and history of low treatment adherence.
	3e	Automatic appointment scheduling and reminders at suitable intervals after new treatment initiation.
	3f	Increased service provision (number and flexibility of appointments) to ensure adequate monitoring as above.
	3g	Further provision of electronic appointments (e.g., video and app) to increase ongoing care access. Must not *replace* overall increased resource but permit patients and clinicians to choose between electronic and face-to-face.
Access to specialist care^a^	4a	Enhanced training programs for primary care physicians to obtain mental health specialist expertise. This is (a) to support people who will not reach secondary care (not replace secondary care) and (b) not to be a wide outreach program as it is considered important that overall GPs remain generalists who are adept across the health spectrum.
	4b	Integrate psychiatrists into primary care, to support GPs and with similar aims as above.
	4c	Equip GPs with increase knowledge of which patients should be referred into secondary mental health care services, and at which stage (see also 4d).
	4d	Service reforms to enable patients’ referrals to secondary care *accepted* (at present refusals are common despite meeting criteria as specified in guidelines, that is, nonresponse to two antidepressants).
	4e	Enhance training programs for doctors into psychiatry (to increase provision in secondary care services).
	4f	Enhance education programs to train psychiatrists and other secondary care practitioners to achieve specialism in mood disorders.
	4g	Implement systems to improve transition for people after discharge from secondary care (e.g., joint working between psychiatric and general physicians, and occasional follow-ups in secondary care after discharge).
	4h	Process for specialist services to establish structured long-term management/follow-up plan for all individuals (incorporating any comorbidities, social support, coping strategies, active involvement of people close to the patient, etc.) to ensure patients do not “fall through the cracks” in the long term.
	4i	Early screening for patients at risk of needing specialist treatment earlier in care pathways (e.g., history of treatment resistance or risk for bipolar disorder).
	4j	Resource input to create more specialist mood disorders centers.

Abbreviation: GP, general practitioner.

a Applies to both secondary and tertiary care, and although most of these items refer specifically to secondary care, these can also apply to secondary care.

When considering the included recommendations, the panel felt that it was important to emphasize that (a) we cannot serve those with depression better without additional funded resources and capacity, and (b) some of the proposed recommendations are already implemented (to a lesser or greater extent) in some localities, but our recommendations are widely (but flexibly, depending on specific system characteristics) applicable. They apply to both single and recurrent episodes. We also highlight that these recommendations must be considered in conjunction with others, for example, policies to encourage help-seeking require increased appointment capacity. Many recommendations require similar actions on the part of policymakers, for example, more funded clinician time, but we have been careful to also incorporate (where possible) other types of provision, for example, digital and self-management tools which may be expandable at lower costs.

*Enhanced diagnosis (n = 4 recommendations):* These aim to both increase the number of patients presenting to services (through improved information provision regarding how to seek help and increased appointment availability) and the likelihood of a primary care practitioner to detect the MDD (longer appointments, integrating self-management e-tools with healthcare records).

*Increasing treatment provision (n = 9):* These primarily aim to support selection of the “right” treatment to each patient, via: evidenced decision-support tools and patient preference (with patients provided evidence-based information about psychological and non-psychological therapy options), removing barriers for patients to enable more time intensive treatments, increased psychological therapy availability, prescribing support tools, shared-care arrangements between clinicians, and adding mental health workers (e.g., nurses) to primary care.

*Continuity of care after treatment (n = 7):* These aim to ensure that suitability of treatments is monitored so that appropriate changes can be detected in a timely manner. Recommendations relate firstly to improved information: self-management tools for patients to optimize adherence, obtain advice/psychoeducation, and monitor benefits and harms; e-tools for clinicians to access this information, indicating a need for reduced or increased follow-up appointments; and measurement-based care within appointments. Second, to increase capacity for follow-up, recommendations included increased appointment provision, electronic follow-up appointments, and automatic scheduling/reminders for regular follow-ups. Finally, this included screening for risk factors that would indicate patients in need of increased, or reduced, follow-up.

*Access to specialist intervention (n = 10):* For the (approximately one third) people who have not responded to primary care treatment, access to more specialist intervention is critical (*n* = 10). Some recommendations related to increased capacity, that is, creation of more specialist mood disorders services; others related to training to increase capacity (training programs for GPs to achieve mental health expertise, general increase of training into psychiatry, and more specific training programs for psychiatrists to achieve mood disorders expertise). Other recommendations pertained to ensuring patients can be accepted into secondary care, that is, through integrating psychiatrists into primary care, equip GPs with knowledge of who should be referred, reforms to ensure referrals are accepted and early screening for patients at risk of later needing specialist treatment. Finally, to facilitate longer-term care, it was recommended to implement systems to improve transition for those being discharged from secondary care and for specialist services to establish long-term care plans).

## Discussion

### Summary of results

Our care pathway analysis provides averages across reviewed data estimating that ~52% of MDD episodes are not diagnosed, and that of people with a diagnosis ~38% are untreated (averages ~65% in samples including undiagnosed cases). Although most individuals with (recurring) MDD access care at some point, delays to treatment average ~4 years (range 1–8 years, with multiple reports averaging 8 years). Most (treated) patients access primary care services with some regularity, although appointments may often not relate to depression. After starting a treatment, ~66% of people are followed up, half of whom may receive guideline accordant follow-up. Overall, ~19% of people with an MDD diagnosis may be able to access secondary/psychiatric care, and in the absence of data, it is expected that a minimal proportion of individuals are able to access specialist mood disorders care.

In summary, many people with symptoms of depression are not able to access or benefit from usual treatments, are not followed up adequately after initial contacts, and cannot access secondary care or specialist services when required. It is emphasized that both treatment gap data and recommendations relate to working age adults, but both child/adolescent and old-age services require additional considerations and examination.

The expert-based consensus reaching process proposed several recommendations that could be used to enhance the management of MDD in services and thus reduce these treatment gaps. Although the recommendations should facilitate gold standard treatment through encouraging care pathway entry and progression (e.g., timely and higher rates of diagnosis [Step 1], treatment [Steps 2–5] and continuity of care), we cannot yet ascertain the precise value of their implementation. There is, however, encouraging evidence that doing so would lessen the MDD burden cost-effectively [22].

### Further considerations of recommendations to minimize treatment gaps

We highlight that the recommendations proposed in this study were intended to be for point of care solutions to minimize the treatment gaps focused on in this study. Thus, we emphasize that firstly they are not comprehensive approaches to improving care for people with depression, and that secondly they do not include recommendations for much needed rigorous research to bolster evidence-based medicine and identify solutions to treatment gaps. One example of a recommendation for which this is particularly relevant is the development of “computerized decision-support tools to help primary care practitioners determine which treatment(s) to prescribe to people with moderate to severe depression”; this was only endorsed at the final stage of the Delphi process despite being proposed early and having high agreement in principle; the panel raised the caveat that these support tools require an evidence base to ensure their effectiveness and feasibility before being introduced as a service improvement measure. This future research must not only examine patient outcomes, but also patient-reported outcomes, specific patient involvement in the research, and consideration of service views, for example, in qualitative studies. A further consideration here is the type or dimension of depression that is being considered; the limitation of not treating depression dimensionally is discussed below, but this is to some extent addressed in the recommendations, many of which are specific to types of depression (e.g., those needing specialist treatment) and/or that account for subtypes (e.g., screening for characteristics indicating need for specific care).

### Care pathway analysis: impedances to interpretation

*Oversensitivity of screening:* Many of the “treatment gap” studies assessed depression using screening tools rather than robust diagnoses, and despite their validation against diagnostic interviews are known to be oversensitive to depression [76, 77]. We therefore might assume that, for example, rates of diagnosis are higher than estimated above.

*Methodological variability:* Evidently, the conclusions that can be drawn regarding the true nature and extent of these treatment gaps are substantially limited by the primary data reviewed. This relates not only to the availability of relevant data, but also the substantial heterogeneity between studies in terms of the populations assessed, procedures employed (e.g., assessment), comparisons undertaken, and designs utilized (e.g., retrospective studies incur risk of recall biases [32, 52]).

*Regional variability:* Additionally, the nature and extent of treatment gaps clearly vary between regions. Differences include the extent of access to medical professionals, service infrastructures, and cultural factors (e.g., relating to stigma, economic, and lifestyle factors). For example, individuals from Italy may have lower help-seeking than other countries [60, 78]. Another example is the definition of secondary care, which is not standardized [72]. There may be differences between countries with a high versus low ratio of the population living in rural (vs. urban) areas, and thus variation in care pathways is also evident within (as well as between) countries. Only six countries were focused on this project, and these are over-representative in terms of economic wealth as well as western European nations. The treatment gaps in many other countries are likely to be significantly greater, and as observed with Hungarian data, there is likely to be far less data available.

*Patient variability:* Certainly, the characteristics of patients included in the studies reviewed impact the extent of treatment gaps. Those with more severe depression are more likely to receive diagnosis, treatment, follow-up, and specialist care [55]. As such, if we had limited our synthesis to those with moderate or severe depression, the treatment gaps would likely have been much smaller, although the quantity of evidence would have been much smaller. There are clearly limitations to treating MDD as a binary category [21]; however, the majority of original investigations have not used a dimensional approach, and because we were already working with a small body of evidence, inclusivity permitted non-exclusion of potentially relevant data. We emphasize, though, that people with more severe depressive symptoms are more likely to be treated adequately. Those with both mental and physical comorbidities may be either more likely to be treated in accordance with guidelines [79] or less likely to [80]. Age also clearly affects diagnosis and treatment of MDD, although the direction of findings is inconsistent [32, 52].

### Methodological considerations

These data were gathered from a wealth of published/unpublished sources. The quantity of data was suboptimal, but also highly heterogeneous, and this is reflected in the wide range of estimates reported for each treatment gap. In addition to the aforementioned limitations, our synthesis was not systematic and is unlikely to be comprehensive, even within the countries focused on. This is also true of the non-systematic review undertaken prior to selecting the treatment gaps of focus. Relatedly, the non-systematic syntheses were inclusive, and although methodological considerations of studies we report on are noted, they were not subject to a formal quality assessment or prioritized so that higher quality studies were emphasized. We were unable to account for key factors in estimating treatment gaps (e.g., severity of depression) and were unable to fully differentiate between time span, or episodes versus lifetime MDD illness, to standardize the type of information reported (e.g., point prevalence versus 12-month prevalence of detection, treatment, or psychiatric service access). We were also unable to determine the precise *reasons* for the manifestation of each treatment gap. These considerations help to explain the substantial heterogeneity observed between studies reported, and we highlight that, as such, the summary results for each treatment gap are speculative to a greater or lesser extent. The initial stage of the project (identifying treatment gaps and synthesizing findings on these) was also limited by not directly involving patient representatives, although patients were involved—critically—in the recommendations stage.

In addition to the treatment gap synthesis, we also acknowledge that the approach used to develop consensus on recommendations that could be implemented in future to lessen these treatment gaps is not comprehensive. The recommendations require extensive resources, and this need is already well known. Nurses and clinical psychologists were not represented on our Delphi expert panel. However, we were careful to consider throughout the process that: (a) a broad scope has benefits, (b) specific recommendations might be more/less feasible in different contexts, and (c) there is likely to be long-term cost benefits to improving care for depression [22]. This work may be used as a starting point for future policy-focused efforts to identify ways that individual healthcare systems can enhance care for depression; in this respect, our linkage of this part of the project to the treatment gap data previously synthesized is a strength, as was our utilization of a standardized methodology.

In this regard also, we would suggest that despite the lack of certainty around the “current treatment gap” estimates, there is a certain amount of consistency across all the data that not enough people with depression are being diagnosed, treated, followed up after treatment, or given specialist care when they need it.

### Future implications

The implications of minimizing treatment gaps are vast. Undetected and untreated illness (and by extension, delays to diagnosis/treatment) increase the risk of illness worsening, and chronic and treatment-resistant depression [11]. Lack of access to appropriate treatment can also lead to individuals with serious clinical needs fluctuating between primary care and emergency or inpatient stays [81]. Thus, access to psychiatric and specialist services is critical, as primary care practitioners do not possess the capacity to deal with difficult-to-treat and high-risk depression (as highly skilled generalists with overflowing caseloads, minimal time, and support). Our development of recommendations that can be used to minimize treatment gaps is not only a novel contribution to academic literature, but exist as a direct link that can be applied, to progress current practice closer to best-practice care for depression.

We conclude that the treatment gaps in depression care are substantial and concerning, from the proportion of people not entering care pathways to those stagnating in primary care with impairing and persistent illness. The impact of optimizing the pathways of care, both on the quality of life and well-being of patients, and on cost-effectiveness parameters, will be addressed in a complementary publication.

## Data Availability

The data that support the findings of this study are available from the corresponding author, R.S., upon reasonable request.

## Supplementary material

For supplementary material accompanying this paper visit https://doi.org/10.1192/j.eurpsy.2022.28. click here to view supplementary material
