# Supplementary material for: Care pathways for people with major depressive disorder: A European Brain Council Value of Treatment study
Source: Eur Psychiatry. 2022 Jun 15;65(1):e36. doi: 10.1192/j.eurpsy.2022.28 (PMC9280921; doi:10.1192/j.eurpsy.2022.28)
Supplement: Supplementary file 1 [file S0924933822000281sup001.docx]

**Supplementary material**

**Supplement 1: Overview of data availability for 6 European countries**

- 1 – Rates of depression not detected (Treatment Gap 1)
- 2 – Time elapsed between onset of depression episode and detection (Treatment Gap 2)
- 3 – Rates of treatment (in primary/community care) for 1) antidepressant drugs and 2) psychological therapies (Treatment Gap 3)
- 4 – Time between detection of depression & treatment of any kind (in primary care) (Treatment Gap 2)
- 5 – Rates/frequency of follow-up contacts after treatment in primary care (Treatment Gap 4)
- 6 – Rates of referral to psychiatric/secondary care services (Treatment Gap 5)
- 7 – Duration/number of contacts within psychiatric/secondary care services (Treatment Gap 5)
- 8 – Rates of referral to specialist mood disorders/tertiary care services (Treatment Gap 5)

|  | **Information requested** | **UK** | **Germany** | **Hungary** | **Sweden** | **Portugal** | **Italy** |
| --- | --- | --- | --- | --- | --- | --- | --- |
| 1 | Rates of depression not detected |  |  |  |  |  |  |
| 2 | Time elapsed between onset of episode & detection **or treatment** (the latter originally separate, merged post-hoc*) |  |  |  |  |  |  |
| 3 | Rates of treatment (in primary/ community care) for 1) antidepressant drugs and 2) psychological therapies |  |  |  |  |  |  |
| 4 | Time between detection of depression and treatment of any kind (in primary care)* |  |  |  |  |  |  |
| 5 | Rates/frequency of follow-up contacts after treatment in primary care |  |  |  |  |  |  |
| 6 | Referral rates to, **&/ contacts within,** secondary care services (the latter originally separate, merged post-hoc*. |  |  |  |  |  |  |
| 7 | Duration/number of contacts within psychiatric (/secondary care) services* |  |  |  |  |  |  |
| 8 | Rates of referral to specialist mood disorders (/tertiary care) services* |  |  |  |  |  |  |

** Originally, the following gaps were identified as separate outcomes; these were merged post-hoc (after completion of the survey) due to lack and overlap of available data: 1) time to diagnosis and time to treatment; 2) rates of referral to secondary care and number/duration of contacts within secondary care; 3) as previously for specialist (tertiary) care for which there was an absence of available data.*

*Green = data available for this country/outcome, yellow = partially relevant data available, red = no data available*

**Supplement 2: In-depth results of treatment gap syntheses**

3.2.1 Treatment gap 1: Rates of depression detection

*Background/international data*

A low prevalence of depression detection is supported by WHO mental health surveys, with 60% of those with MDD not seeking treatment and a further 24% seeking but not receiving help (1). However, of the 57% of patients stating a need for treatment, 70% had at least 1 contact with services; therefore, this household survey may estimate the true rate of diagnosable MDD detection as lying between 40-70% (1). A Canadian survey of individuals living with chronic diseases found that of 608 participants reporting a major depressive episode, 153 had been in contact with primary care services regarding their depression, suggesting a detection rate of about 25% (2). A 2009 meta-analysis synthesised 41 studies of primary care (GP) sensitivity at detecting depression internationally and reported that depression was correctly detected in 47% (95% CI 42 to 53) of cases. Their findings indicated that detection sensitivity varied greatly between the studies from 6.7% (Hungary) to 78.8% (Netherlands) and was heterogeneous within countries (UK studies ranging between 20 to 63%). The vast heterogeneity may be partly attributable to the lack of a standardised method of determining true and false cases and a lack of adjustment for depression severity (3). A subsequent international meta-analysis (2011) of clinicians’ ability to identify depression in primary care assessed the specificity (proportion of ‘false’ MDD cases correctly identified) and overall accuracy (proportion of total correct results) in addition to sensitivity (proportion of ‘true’ MDD cases correctly identified). They reported that of the countries assessed, UK studies had the lowest sensitivity (46%; 95% CI 28 to 64) but high uncertainty (with insufficient data and a lower overall prevalence than other countries), while Italy had the highest sensitivity (64%; 95% CI 44 to 82) and other countries non-significantly lower than Italy. Incorporating sensitivity and specificity, overall accuracy at detecting depression indicated that Italy (84%; 95% CI 82 to 85) and the Netherlands (82%; 95% CI 80 to 84) was better than the US (74%; 95% CI 71 to 77) and Australia (67%; 95% CI 65 to 69) (4).

*Portugal*

The Portuguese National Mental Health Survey (2008-2009, as part of the World Mental Health Survey initiative) found that the 12-month prevalence of MDD in the general population was 6.8% (diagnosed using the CIDI DSM-IV interview) and for depressive symptoms (using the PHQ9 screening tool) was 10% (5,6). These estimates likely included undetected depression. National data from the Portuguese Ministry of Health (2016-2017), using information from primary care medical records – indicating only detected cases - finds the 12 month prevalence of depressive symptoms reported in services to be 9.3% (7). Although taken together these data would appear to suggest a very high level of depressive symptom detection in Portugal, this conclusion would be highly speculative since the latter report does not include diagnosed cases, and potentially the prevalence of depressive symptoms may have increased between 2008-2017.

*Sweden*

Estimates of *diagnosed* MDD have ranged from 1% (1990’s study of 374 primary care patients) (8), 1.1% (2002 health records database study of ~600,000 people assessing a range of depressive disorders) (9), 4% (2018 national public health survey of ~16,600 people) (10) to 8.5% (2001 primary care study of 1348 patients) (11). Estimates of true MDD point prevalence have ranged from 4% (1990’s study of 374 primary care patients) (8) to 6.2% (2005 primary care study of 1392 patients) (12). Although 4% had a diagnosis in the 2018 National Public Health survey (n~16,600), rates of self-reported severe anxiety were 7%, severe fatigue 9%, severe insomnia 7%, severe stress 4% and reduced psychological wellbeing 17% (10). These data may indicate a higher depression prevalence rate more in line with the above estimates, potentially around 7%. Although one of the above studies reported as low a detection rate as 25% (1990’s study of 374 primary care patients) (8), it might be speculated that from the larger and/or more recent studies the true point prevalence of diagnosed MDD in Sweden is around 4%, giving an indicative detection rate of approximately 57%, albeit with a large potential margin of error.

*UK*

The UK’s 2014 national Adult Psychiatric Morbidity Survey (APMS) investigated a representative household sample. When depression was combined with anxiety disorders (using the umbrella term common mental disorder (CMD)), its point prevalence was 17%. The most common CMD was a ‘not otherwise specified’ anxiety/depression category (7.8% of the entire sample) in addition to 3.3% of all adults meeting clear substantive criteria for current MDD. 35% of men disclosed having some lifetime experience of CMD, 20% having received a diagnosis; 51% of women disclosed a lifetime CMD experience, with 35% having received a diagnosis. 49% of those with a CMD had been diagnosed as such within the last year (13). More specifically examining detection of major depressive episodes (MDEs), a cohort study of 179 consecutive primary care patients (1997-2001) reported that 49% met criteria for a CMD at study entry, of whom only 39% had been diagnosed although a further 25% were diagnosed in the subsequent three years (totalling 64% detection over three years). The study reported that a high proportion of those without a CMD diagnosis nevertheless had severe symptoms of depression and functional impairments (14). A similar study comparing GP and patient ratings reported a point prevalence of depression between 13-16% with GPs detecting depression with a sensitivity of 30-33%; GPs detected depression in 49% of individuals at a first phase and 35% in a second phase three years later (15).

*Germany*

A primary care study published in 2002 reported correct identification of 55% of depressed individuals although only 21% had a formal diagnosis of ‘definite depression’ (16). A large medical records study (covering 87% of the population) reported the 12-month prevalence of *diagnosed* depression to increase from 12.5% to 15.7% between 2009 and 2017 (17). This was reduced to 5.6% (2010) and 10.1% (2017) when excluding unspecified diagnostic codes for depressive disorder. 2009-2012 population surveys, thus including *undiagnosed* cases, estimated a stable MDD prevalence approximating 7.4% (not including unspecified depression subtypes) (18). If speculative comparing 2010 data between these two reports, it might be estimated that ~75% of MDEs are detected (5.6/7.4). The suggestion of an increase in diagnosed depression in the last decade may be attributable to increased overall prevalence or increased detection rates. An older survey-based study (1998-2010) suggested that help-seeking increased (not seeking help was reduced from ~ 62% to 57% during this time frame) (19). In recent years it has even been purported that over-diagnosis of MDD has increased (17).

*Italy*

The international meta-analysis of MDD detection rates included four Italian studies spanning from 1994 to 2009 with a sample of 2836 subjects, and found the diagnostic sensitivity of Italian GPs to be 64% (4,20). A study involving more than 20850 subjects using data from the Italian behavioural risk factor surveillance system (based on telephone interviews) found that 47% of people with depressive symptoms did not seek help (21). Help-seeking may be even lower; other reports suggest that 80% of the Italian population suffering from depressive symptoms do not seek help (22). Although the diagnostic sensitivity of GPs appears to be around two-thirds of patients, this may not adequately account for low levels of help-seeking and taken together the data may speculatively suggest that the true rate of detection depression might be as low as one-third of people meeting MDD criteria.

*Hungary*

The MDD prevalence rate in Hungary is estimated to be 15.1% lifetime and 7.1% over 12 months. The 2009 international meta-analysis of GP sensitivity at detecting depression reported the rates in Hungary to be lower than any other country, with a sensitivity of 6.7%, although these data need verifying (3).

3.2.2 Treatment gap 2: Time to diagnosis or treatment

*Background/international data*

Data from a 2014 survey of 3213 individuals living with mood and/or anxiety disorders in Canada suggested that depression was diagnosed more than one year after symptom onset in 61% (n = 1882) of respondents and the delay to diagnosis was more than five years in approximately 30% of individuals. The reported median time to onset was 4.4 years (2). This study’s findings were based on individuals’ self-reports and may have been influenced by recall bias.

Data across 11 countries from a 2007 self-administered questionnaire for patients with an anxiety or mood disorder found that 40% had sought treatment in the first year of symptom onset but the median delay of help-seeking was 8 years. It should be noted that this was a retrospective study of CMDs, not specific to MDD (23). Similar results were found in a more recent survey of lifetime psychiatric diagnoses in the adult Argentinean population including 446 participants with an MDD diagnosis. 32% had received treatment in the year of illness onset but the median delay was 8 years. Interestingly, all patients with persistent MDD symptoms at some point in their lifetime sought treatment. Recall bias due to the study’s retrospective design should be considered when interpreting these findings (24). Data from a US national survey of comorbidities from 2001-2003 (n = 9282) also reported that 37% of participants with MDD had sought treatment within a year of illness onset and a median delay to treatment of 8 years. They also found that almost all had a lifetime treatment contact (88-94%) (25). Finally, an analysis of data from 2,419 Belgian participants with various mood disorders (including bipolar as well as unipolar depression) in the European study of epidemiology of mental disorders (ESEMeD) study found that 94% of those with mood disorders seek treatment at some point during the lifetime (higher than for other conditions assessed) but only 48% seek help within the first year of experiencing a mood disorder. The average (median) delay between first illness onset and the first treatment contact was 1 year (26).

*Portugal*

Although Portugal’s National Mental Health Survey reported some data related to treatment rates and delay, these were not considered sufficiently reliable for inclusion in the report (5,6).

*UK*

Unpublished data from a naturalistic study of individuals referred into the Improving Access to Psychological Therapies (IAPT national programme) service in South London (27) indicates a mean of 8 years between mental illness onset and first contact with healthcare services (range 0 – 45 years), with 18% having a delay-time of less than 1 year. However, these (similar to above) are lifetime estimates. Data focused within a depressive episode in a sample of 178 people with TRD indicated the time from episode onset to antidepressant treatment as being over two years in 43% of the sample, 1-2 years in 13%, 4-12 months in 15%, 0-3 months in 13%, and 17% had relapsed while undergoing treatment (28). According to Mental Health Statistics for England in 2018/2019, the average waiting time to begin IAPT talking therapy treatment was 20 days and 99% of people initiated treatment within 18 weeks (29). However, it has been reported that IAPT maintain low waiting times by offering few therapy sessions or giving access to online self-help packages, and many patients are not accepted for therapy if their illness is considered severe (despite commonly not being able to access therapy via secondary mental health services). Furthermore, an investigation by the British Medical Association in 2018 revealed severe delays to psychological treatments in the NHS in general; despite limited recording of this data, almost 4000 patients waited over six months to begin a talking therapy and 1500 waited longer than one year (30). A mental health charity survey (Mind, 2018) in England and Wales reported that 33% of 8000 respondents had waited >6 days for their most recent primary care appointment (not specific to depression) and 12% had been unable to make an appointment in the last year (31).

*Germany*

A survey undertaken at a German convention for people with depression found that, of 418 respondents (mainly patients and their relatives), ~66% experienced a delay of more than 3 months between symptom onset and first treatment, with approximately the same proportion of individuals receiving a diagnosis initially from a specialist as opposed to primary care. Therefore, it should be considered that the average delay to treatment in people *only* seen in primary care is likely substantially higher than this. Indeed approximately 25% of the sample reported more than 3 years between symptom onset and treatment initiation. To summarise, of the total sample just over one third received treatment within 3 months of episode onset, one third between 3 months and 3 years, and one quarter over 3 years (32).

*Italy*

One multicentre study (n = 1140) reported a duration of undetected illness to be <6 months in 64% of MDD patients (33). A further study of 181 MDD patients reported an average duration of untreated illness of 3.25 years (34). Both of these studies were drawn from patients attending secondary care services and therefore were not representative of a whole depressed population, and it is reasonable to suppose that the vast majority of patients referred to psychiatric services have already been prescribed a drug treatment in primary care and may have had more severe depression initially than average (35). In primary care, it was estimated that only 12.9% of untreated patients considered likely to benefit from antidepressants started a new drug treatment (35).

3.2.3 Treatment gap 3: Rates of treatment with pharmacological and psychological therapies

*Background/international data:*

SSRI antidepressants are the most common first-line treatment for depression (14,15). Data from a large US study (2014-2019) of four national claims databases (n=269,668) reported rates of SSRI treatments in 36-58% of MDD-diagnosed individuals, but approximately one third of diagnosed patients were not receiving treatment (30-36% in 3/4 databases and 52% in the fourth database (36). Data from a USA household survey (n=46417) estimated only 28.7% of patients to be receiving any treatment, although this sample comprised those screening positively for depression and therefore includes undiagnosed individuals but may contain some false positive depression cases (depression prevalence reported to be 8.4%). Of the treated participants 23% received psychotherapy, 87% monoaminergic antidepressants and 5-15% each of other medication classes (e.g. anxiolytics, antipsychotics, mood stabilisers) (37). The aforementioned ESEMeD study reported that of those with a mood disorder, 15% did not receive any treatment, 38% were treated with medication, 33% combination psychological and psychotropic treatment and 14% psychological monotherapy treatment (38) although this may be an overestimate for MDD, as it also included those with a bipolar disorder. A Canadian survey of individuals living with chronic diseases found that, despite a low diagnosis rate of depression (~25%), 65% of those diagnosed had received some form of treatment (from any practitioner) (2). International data from the large WHO mental health surveys (2017) indicated that only 16.5% of people scoring positively for MDD received *adequate* treatment (sufficient course of pharmacotherapy or psychotherapy with regular healthcare contacts) (1). This proportion might be particularly low since most studies report rates of individuals receiving any treatment rather than requiring a degree of adequacy, and these surveys might have included more individuals with either subclinical or clinical but undetected depression (especially as a high proportion did not report a perceived need for treatment, although the 12-month prevalence was 4.6%, lower than other population estimates).

*Portugal*

From data collected as part of the Portuguese National Mental Health Survey (2008-2009), 55.5% of individuals with 12-month MDD (N=293) were receiving any professional treatment for “emotions, nerves, mental health, or use of alcohol or drugs”; 44.5% were not receiving treatment. 38.4% people with diagnosed MDD received treatment in primary care, most commonly sedatives (40%), followed by antidepressants (38%), tranquilisers (32%) and antipsychotics (2.2%). For psychological therapies, data are only available for lifetime experience with psychological therapy lasting >30 minutes with any type of professional; among individuals with 12-month MDD the rate of therapy was 37.7% (5,6).

*Sweden*

National statistical databases indicated the total prescription rates for antidepressant medication (all ages) ranged from 1,004,421 (2018) to 1,042,162 (2019) and for SSRI’s alone 639,910 (2018) and 652,765 (2019). The total number of dispatched packets of antidepressants exceeded 6.7 million in each year (39). The population of Sweden above the age of 15 (below which minimal antidepressant use would be expected) is approximately 8.45 million, which suggests that around 12% are taking antidepressants. This appears high, given the 12-month MDD prevalence estimate of 6-8%, however antidepressants are also frequently used by people with other illnesses, especially anxiety disorders (at least as common as MDD), and also include those currently with MDD in remission. Thus, little interpretation can be made from these data. Another study, referenced previously, which identified a particularly low rate of depression detection, reported that 47% of people with diagnosed depression were *offered* appropriate treatment, usually SSRI antidepressants and appointment(s) with a medical social worker (8).

*UK*

A 2005 observational study in primary care reported that 73% of patients screening positively for depression were not receiving any treatment (in the presence also of a low MDD diagnosis rate). Of the total sample recorded as having depression, GPs detected illness in 35-49%, offered antidepressants to 8-22% and referred 7-16% for counselling. It was noted that despite the low rate of antidepressant offering, antidepressants were discussed with 44% of diagnosed patients (15). Similarly, the England Adult Psychiatric Morbidity studies found that 77% (2000) and 76% (2007) of individuals with a CMD did not receive any pharmacological or psychological treatments (40). The untreatment rate was slightly lower in the 2014 survey (62%; 52% in those with severe illness and 61% of those with moderate illness) and the authors also reported treatment rates in those with MDD as being 55% pharmacological and 23% psychological. In addition, almost 2% of participants with CMD reported having asked for, but not received, treatment and this rate increased to 10% of those categorised as having severe CMD symptoms (13). Updated data from prescription records demonstrates that approximately 6.5 million antidepressant prescriptions are made per month in England, more than half of which are for SSRI’s (41) and data from the Health Foundation previously found a 165% increase in antidepressant prescriptions from 1998 to 2012, which was suggested to reflect an increased treatment rate, rather than increased illness prevalence rates (42). Psychological therapy provision also appears to have increased; English Mental Health Statistics from 2018/2019 report a referral rate to IAPT services of 1.6 million (for CMD) of which 1.1 million began treatment and one third of those referred completing a course of therapy (average 7 sessions) (29). 2019/2020 data replicate this and suggest that approximately 52% of treatment completers are subsequently referred on to a further recovery program (43). A UK study of people with TRD (n=178), and therefore all of whom had received >2 medication treatments within their MDD episode, 68% had previously accessed a psychological therapy but this reduced to 53% when only counting *adequate* psychological therapies according to treatment guidelines. Notably, only 20% of the sample had received adequate treatment (psychological or pharmacological) within 2 years of episode onset (28). Another UK primary care study of 235 people with early-stage TRD (who were thus all medicated) found that over a 12-month period, less than 25% had received any talking therapy during the year (and only 2.8% received cognitive behavioural therapy (CBT) within the first 6 months). Interestingly, 1.5% reported having an inpatient stay and 1.5% had been to emergency department(s) for their mental health (44). An examination of anonymous medical record data in three UK cities from 2006-2007 (covering 2294 patients across 38 primary care practices) found that 79% of *diagnosed* depressed patients were prescribed antidepressants and when excluding those noted as having only mild symptoms the rate increased to 87% (45). A primary care cohort study (2002) reported that, notwithstanding a low MDD diagnosis rate, 68% were treated with antidepressants (14). Both of the latter two studies report high treatment rates compared to other estimates; this may be attributable to only examining patients who not only had MDD diagnosis but were also well characterised and being conducted prior to national psychological therapy service implementation. While IAPT has considerably increased non-pharmacological treatment rates, it is possible that IAPT referrals (not necessarily equating to therapy access) reduced to some extent antidepressant medication prescriptions.

*Italy*

A primary care study (n=1413) reported that of patients with MDD considered to benefit from antidepressants, only 39% were treated with them (46). This finding was replicated in a similar investigation (n=2093) where 21% of diagnosed patients likely to benefit from antidepressants were treated as such. The proportion of all depressed patients receiving non-pharmacological interventions (non-exclusive to the above medication rates) was 37%, although most patients received counselling and only 16% underwent a psychotherapy. Overall, 29% of these patients were not receiving any psychological or pharmacological treatment (35).

*Germany*

In a (2000) primary care study (MDD prevalence 11%), only 4% of diagnosed MDD patients had not received any intervention, compared to 12% of those with depression and had a different psychiatric diagnosis) (16).

*Hungary*

It has been reported that approximately 40-45% of (detected) depressed individuals receive treatment (47,48).

3.2.4 Treatment gap 4: Rates and frequency of follow-up contacts after treatment/continuity of care.

*Background/international data*

A study of USA national health plan (2000-2004) data reported follow-up visits in people diagnosed with MDD (n=4102) after initiating antidepressant treatment to average 2.6 visits within 3 months overall. The rate for those whose medication was prescribed by a psychiatrist was 3.8 compared to primary care 2.0. The authors summarised that 31% of the sample had been followed-up in accordance with best-practice guidelines (>3 appointments in 3 months) and this was higher (52%) for those treated by a psychiatrist compared with GP (19%) (49). A cohort of US veterans (n=2178) similarly reported that 26% of patients received adequate follow-up care (defined as above) after treatment initiation although 33% did not have any follow-up visits in this period (the first follow-up averaged 28 days after initiation) (50). A third American study reported that half of participants (n=274) received at least 1 follow-up visit within three months of primary care treatment initiation (51).

*Portugal*

The Portuguese national mental health survey (2008-2009) data indicated that those receiving treatment in primary care had an average of 3.27 (mean) and 2.00 (median) general medical consultations in 12 months i.e. on average one appointment every 4-6 months (5,6).

*Italy*

A nationwide study of 1896 participants attending primary care clinics (n=250 MDD), reported that over a 6-month follow-up, 60% of depressed patients attended appointments (any reason) more than once a month but as only 74% of depressed participants had received an MDD diagnosis, these data include one quarter of depressed patients without a diagnosis (20).

*UK*

National statistics have reported that 64% of patients with newly diagnosed depression had a primary care review <56 days after initial diagnosis (52), broadly aligning with NICE guidelines. Similarly, a retrospective primary care medical records study found that 78% (of 279 patients, 2002-2004) receiving treatment after MDD diagnosis were offered within 28 days of treatment start (53). A second examination of anonymous medical record data in three UK cities (excluding individuals with only minimal/mild symptoms; n=1709), reported that 67% attended a primary care follow-up visit within 4 weeks of treatment initiation (45). A primary care study of 235 people with TRD examined service use over a one-year period (2008-2010) and reported an average of 4 GP visits in 6 months (44), although other evidence supports those with TRD having significantly higher healthcare utilisation than other depressed populations across multiple European countries studied (54).

3.2.5 Treatment gap 5: Access to secondary (or psychiatric) care services.

*Background/international data*

A USA household survey (8% MDD prevalence, of which only 29% received treatment) found that 24% of treatment was managed through a psychiatrist, an almost equivalent rate to the prevalence of TRD (73% treated in primary care) (37). Thus, it might be reasonable to extrapolate that most people who are inadequately treated through primary care can access specialist support in line with their needs. This rate is similar to that reported in a Seattle-based prospective cohort study (2002; n=942), where 23% of people with depressive symptoms in private primary care were referred to a mental health specialist and 38% saw a mental health specialist (regardless of referral) over the upcoming 6 months (55). The 1998-1999 Canadian national population health survey also reported that of diagnosed MDD cases, 26% had been referred to mental health specialists (psychiatrist or psychologist) (2).

The Netherlands Study of Depression and Anxiety (NESDA; n=478) found that 58% of the sample with an MDD diagnosis had been referred to mental health care, but about half of these (54%; equating to approximately one quarter of the depressed population) were to secondary mental health care (56). However, one of the study’s recruitment sources had been through secondary care which may have inflated estimates.

Perhaps most significantly, almost all of the estimates in this section relate to referrals rather than actual visits or treatment changes as a result of accessing specialist care, and it has been reported that only about three quarters of referrals manifest in secondary care contacts (55).

*Portugal*

The Portuguese national mental health survey (2008-2009) indicate that the proportion of individuals with MDD receiving any specialized mental health treatment (psychiatrist or psychologist) within 12 months was 28.1% and 21.9% received psychiatric treatment in the previous year. Lifetime referral rate for these individuals (i.e. answered ‘yes’ to “Did a medical doctor other than a psychiatrist *ever* recommend that you go to a mental health specialist, clinic or program”?) was 22.1% (5,6). This may mean that other individuals under secondary care, within the previous year (specified above) were not first recommended.

*Italy*

0.3% of the Italian population attends psychiatric secondary care public services with a diagnosis of mood disorder (point prevalence), with an annual prevalence rate of mood disorder of ~3%. Although this data may be now outdated, the estimated coverage of secondary care services is 7.6% (57). It is reasonable to suppose that discounting bipolar disorder (which is associated with higher service use, particularly in secondary care) and limiting the sample to MDD only, this rate would be markedly lower. A study of referrals to psychiatric secondary care public services in Italy reported that only 18 out of 224 participants with depressive symptomatology had accessed secondary care (8%) although this includes those without an MDD diagnosis and may be an underestimation (35). A 2009 survey on psychiatric service use in a single region found that, following discharge from an acute psychiatric ward, only 18% of patients with an MDD diagnosis waited more than a week for a psychiatric appointment in secondary care services. However, only 16% of these patients received at least one visit per month over the next six months, only 60% continued taking antidepressants for >6 months and only 38% for >12 months. It is therefore reasonable to suppose that they continued follow-up visits for an even shorter period, in some cases not attending appointments before discontinuing medications (57).

*UK*

Examination of anonymous medical record data in three UK cities (2006-2007) reported that 23% of individuals screened for depression in standard care were referred to ‘specialist services’, although this was defined broadly, including a counselling service (14%), primary care mental health worker (5%), psychology service (2%), social services (0.5%) and psychiatry (5%). This study was conducted before the implementation of IAPT which has considerably increased psychological therapy provision. These data indicate a rate of about 5% of patients referred to a psychiatric secondary care service (45). In the aforementioned MDD primary care study, 21% of MDD-diagnosed patients were referred to psychiatric services (secondary care) over three years follow-up, but this assessed referral rather than actual contact rates. This study had a small sample and it is possible that GP’s were aware that their patients were being monitored in a depression treatment cohort study (14). In the latter primary care study of people with early-stage TRD (2008-2010) only 6.1% had received treatment in NHS outpatient or community mental health clinics (44). Data from the aforementioned TRD study (28) indicated that 44% of patients had access to secondary care (although this was substantially lower in London (23%) than Newcastle (46%) or Oxford (72%)) and it should be noted that 1) this may have included individuals with a privately-accessed psychiatrist, 2) psychiatrist contacts may have been related to other psychiatric comorbidities and 3) secondary care referrals were one method of study recruitment, all of which may have inflated this estimate (28).

*Germany*

In a (2000) primary care study (MDD prevalence 11%), 12% of diagnosed patients had been referred to a mental health specialist; this was compared to 10% of those with depression but who had a different psychiatric diagnosis (16). This study did not report on actual secondary care contacts, however other evidence suggests that more individuals are seen for common mental disorders within secondary care in Germany than many other countries (58) despite the limited data available for this outcome.

1. Thornicroft G, Chatterji S, Evans-Lacko S, Gruber M, Sampson N, Aguilar-Gaxiola S, et al. Undertreatment of people with major depressive disorder in 21 countries. Br J Psychiatry. 2017 Feb;210(2):119–24.

2. Ricky C, Siobhan O, Nawaf M, Elliot M. G. Factors associated with delayed diagnosis of mood and/or anxiety disorders. Health Promot Chronic Dis Prev Can Res Policy Pract. 2017 May;37(5):137–48.

3. Mitchell AJ, Vaze A, Rao S. Clinical diagnosis of depression in primary care: a meta-analysis. Lancet Lond Engl. 2009 Aug 22;374(9690):609–19.

4. Mitchell AJ, Rao S, Vaze A. International comparison of clinicians’ ability to identify depression in primary care: meta-analysis and meta-regression of predictors. Br J Gen Pract J R Coll Gen Pract. 2011 Feb;61(583):e72-80.

5. Xavier M, Baptista H, Mendes JM, Magalhães P, Caldas-de-Almeida JM. Implementing the World Mental Health Survey Initiative in Portugal – rationale, design and fieldwork procedures. Int J Ment Health Syst. 2013 Jul 9;7:19.

6. Antunes A, Frasquilho D, Azeredo-Lopes S, Neto D, Silva M, Cardoso G, et al. Disability and common mental disorders: Results from the World Mental Health Survey Initiative Portugal. Eur Psychiatry. 2018 Mar;49:56–61.

7. Relatório do Programa Nacional para a Saúde Mental 2017 [Internet]. [cited 2020 Aug 3]. Available from: https://www.dgs.pt/em-destaque/relatorio-do-programa-nacional-para-a-saude-mental-2017.aspx

8. Bodlund O. [Anxiety and depression as a hidden problem in primary health care. Only one case in four identified]. Lakartidningen. 1997 Dec 3;94(49):4612–4, 4617–8.

9. Lejtzén N, Sundquist J, Sundquist K, Li X. Depression and anxiety in Swedish primary health care: prevalence, incidence, and risk factors. Eur Arch Psychiatry Clin Neurosci. 2014 Apr;264(3):235–45.

10. PxWeb - välj tabell [Internet]. [cited 2020 Aug 3]. Available from: http://fohm-app.folkhalsomyndigheten.se/Folkhalsodata/pxweb/sv/B_HLV/B_HLV__dPsykhals/

11. Allgulander C, Nilsson B. [A nationwide study in primary health care: One out of four patients suffers from anxiety and depression]. Lakartidningen. 2003 Mar 6;100(10):832–8.

12. Nordström A, Bodlund O. Every third patient in primary care suffers from depression, anxiety or alcohol problems. Nord J Psychiatry. 2008 Jan 1;62(3):250–5.

13. Adult Psychiatric Morbidity Survey: Survey of Mental Health and Wellbeing, England, 2014 - NHS Digital [Internet]. [cited 2020 May 11]. Available from: https://webarchive.nationalarchives.gov.uk/20180328140249/http://digital.nhs.uk/catalogue/PUB21748

14. Kessler D, Bennewith O, Lewis G, Sharp D. Detection of depression and anxiety in primary care: follow up study. BMJ. 2002 Nov 2;325(7371):1016–7.

15. Kendrick T, King F, Albertella L, Smith PW. GP treatment decisions for patients with depression: an observational study. Br J Gen Pract. 2005 Apr 1;55(513):280–6.

16. Wittchen H-U, Pittrow D. Prevalence, recognition and management of depression in primary care in Germany: the Depression 2000 study. Hum Psychopharmacol. 2002 Jun;17 Suppl 1:S1-11.

17. Steffen A, Thom J, Jacobi F, Holstiege J, Bätzing J. Trends in prevalence of depression in Germany between 2009 and 2017 based on nationwide ambulatory claims data. J Affect Disord. 2020 Jun 15;271:239–47.

18. Bretschneider J, Janitza S, Jacobi F, Thom J, Hapke U, Kurth T, et al. Time trends in depression prevalence and health-related correlates: results from population-based surveys in Germany 1997–1999 vs. 2009–2012. BMC Psychiatry [Internet]. 2018 Dec 20 [cited 2020 Sep 17];18. Available from: https://www.ncbi.nlm.nih.gov/pmc/articles/PMC6302526/

19. Brandstetter S, Dodoo-Schittko F, Speerforck S, Apfelbacher C, Grabe H-J, Jacobi F, et al. Trends in non-help-seeking for mental disorders in Germany between 1997–1999 and 2009–2012: a repeated cross-sectional study. Soc Psychiatry Psychiatr Epidemiol. 2017 Aug 1;52(8):1005–13.

20. Menchetti M, Murri MB, Bertakis K, Bortolotti B, Berardi D. Recognition and treatment of depression in primary care: Effect of patients’ presentation and frequency of consultation. J Psychosom Res. 2009 Apr 1;66(4):335–41.

21. Binkin N, Gigantesco A, Ferrante G, Baldissera S. Depressive symptoms among adults 18–69 years in Italy: results from the Italian behavioural risk factor surveillance system, 2007. Int J Public Health. 2010 Oct 1;55(5):479–88.

22. istituto nazionale di statistica. Mental health at various stages of life [Internet]. 2018. Available from: https://www.istat.it/it/files//2018/07/Mental-health.pdf

23. Christiana JM, Gilman SE, Guardino M, Mickelson K, Morselli PL, Olfson M, et al. Duration between onset and time of obtaining initial treatment among people with anxiety and mood disorders: an international survey of members of mental health patient advocate groups. Psychol Med. 2000 May;30(3):693–703.

24. Stagnaro JC, Cía AH, Aguilar Gaxiola S, Vázquez N, Sustas S, Benjet C, et al. Twelve-month prevalence rates of mental disorders and service use in the Argentinean Study of Mental Health Epidemiology. Soc Psychiatry Psychiatr Epidemiol. 2018 Feb;53(2):121–9.

25. Wang PS, Lane M, Olfson M, Pincus HA, Wells KB, Kessler RC. Twelve-month use of mental health services in the United States: results from the National Comorbidity Survey Replication. Arch Gen Psychiatry. 2005 Jun;62(6):629–40.

26. Bruffaerts R, Bonnewyn A, Demyttenaere K. Delays in seeking treatment for mental disorders in the Belgian general population. Soc Psychiatry Psychiatr Epidemiol. 2007 Nov;42(11):937–44.

27. Hepgul N, King S, Amarasinghe M, Breen G, Grant N, Grey N, et al. Clinical characteristics of patients assessed within an Improving Access to Psychological Therapies (IAPT) service: results from a naturalistic cohort study (Predicting Outcome Following Psychological Therapy; PROMPT). BMC Psychiatry. 2016 Feb 27;16:52.

28. Day E, Shah R, Taylor RW, Marwood L, Nortey K, Harvey J, et al. A retrospective examination of care pathways in individuals with treatment-resistant depression. BJPsych Open [Internet]. 2021 May [cited 2021 Sep 27];7(3). Available from: https://www.cambridge.org/core/journals/bjpsych-open/article/retrospective-examination-of-care-pathways-in-individuals-with-treatmentresistant-depression/22D554A17531E258F8E93CF9A94897B4

29. Baker C. Mental health statistics: prevalence, services and funding in England. 2020 Sep 17 [cited 2020 Sep 17]; Available from: /research-briefings/sn06988/

30. Scavone F. The devastating cost of treatment delays [Internet]. The British Medical Association is the trade union and professional body for doctors in the UK. [cited 2020 Sep 28]. Available from: https://www.bma.org.uk/news-and-opinion/the-devastating-cost-of-treatment-delays

31. MIND. One in three say mental health deteriorates while waiting for GP appointment [Internet]. 2018 [cited 2020 Sep 28]. Available from: https://www.mind.org.uk/news-campaigns/news/one-in-three-say-mental-health-deteriorates-while-waiting-for-gp-appointment/

32. Dietrich S, Mergl R, Rummel-Kluge C. Von den ersten Symptomen bis zur Behandlung einer Depression. Wann und bei wem suchen Menschen mit Depression Hilfe? Welche Rolle spielt Stigmatisierung? Psychiatr Prax. 2017 Nov;44(8):461–8.

33. Aguglia E, Biggio G, Signorelli MS, Mencacci C, Steering Committee on behalf of the STIMA-D Investigators. Italian Study on Depressive Disorders (STudio Italiano MAlattia Depressiva, or STIMA-D): a nationwide snapshot of the status of treatment for major depression. Pharmacopsychiatry. 2014 May;47(3):105–10.

34. Altamura AC, Buoli M, Albano A, Dell’Osso B. Age at onset and latency to treatment (duration of untreated illness) in patients with mood and anxiety disorders: a naturalistic study. Int Clin Psychopharmacol. 2010 May;25(3):172–179.

35. Balestrieri M, Carta MG, Leonetti S, Sebastiani G, Starace F, Bellantuono C. Recognition of depression andappropriateness of antidepressant treatment in Italian primarycare. Soc Psychiatry Psychiatr Epidemiol. 2004 Mar 1;39(3):171–6.

36. Kern DM, Cepeda MS, Defalco F, Etropolski M. Treatment patterns and sequences of pharmacotherapy for patients diagnosed with depression in the United States: 2014 through 2019. BMC Psychiatry. 2020 Jan 3;20(1):4.

37. Olfson M, Blanco C, Marcus SC. Treatment of Adult Depression in the United States. JAMA Intern Med. 2016 Oct 1;176(10):1482–91.

38. Alonso J, Angermeyer MC, Bernert S, Bruffaerts R, Brugha TS, Bryson H, et al. Use of mental health services in Europe: results from the European Study of the Epidemiology of Mental Disorders (ESEMeD) project. Acta Psychiatr Scand Suppl. 2004;(420):47–54.

39. Statistikdatabaser - Läkemedelsstatistik - Val [Internet]. [cited 2020 Aug 3]. Available from: https://sdb.socialstyrelsen.se/if_lak/val.aspx

40. (PDF) Adult psychiatric morbidity in England, 2007: Results of a household survey [Internet]. ResearchGate. [cited 2020 Sep 28]. Available from: https://www.researchgate.net/publication/266299241_Adult_psychiatric_morbidity_in_England_2007_Results_of_a_household_survey

41. EBM lab. Antidepressant drugs [Internet]. OpenPrescribing.net. 2020 [cited 2020 Sep 28]. Available from: https://openprescribing.net/bnf/0403/

42. Spence R, Roberts A, Ariti C, Bardsley M. Focus on: Antidepressant prescribing trends in the prescribing of antidepressants in primary care. 2014;

43. Psychological Therapies, Annual report on the use of IAPT services 2018-19 [Internet]. NHS Digital. [cited 2020 Sep 28]. Available from: https://digital.nhs.uk/data-and-information/publications/statistical/psychological-therapies-annual-reports-on-the-use-of-iapt-services/annual-report-2018-19

44. Management of treatment-resistant depression in primary care: a mixed-methods study | British Journal of General Practice [Internet]. [cited 2020 Sep 28]. Available from: https://bjgp.org/content/68/675/e673

45. Kendrick T, Dowrick C, McBride A, Howe A, Clarke P, Maisey S, et al. Management of depression in UK general practice in relation to scores on depression severity questionnaires: analysis of medical record data. BMJ [Internet]. 2009 Mar 19 [cited 2020 Sep 28];338. Available from: https://www.bmj.com/content/338/bmj.b750

46. Bellantuono C, Mazzi MA, Tansella M, Rizzo R, Goldberg D. The identification of depression and the coverage of antidepressant drug prescriptions in Italian general practice. J Affect Disord. 2002 Oct 1;72(1):53–9.

47. Zoltán R. A depressziók kezelése és a hazai öngyilkossági halálozás kapcsolata – fókuszban a 2007-es egészségügyi reform hatása. Neuropsychopharmacol Hung. :10.

48. Rihmer Z, Gonda X, Kapitany B, Dome P. Suicide in Hungary-epidemiological and clinical perspectives. Ann Gen Psychiatry. 2013 Jun 26;12(1):21.

49. Chen S-Y, Hansen RA, Farley JF, Gaynes BN, Morrissey JP, Maciejewski ML. Follow-Up Visits by Provider Specialty for Patients With Major Depressive Disorder Initiating Antidepressant Treatment. Psychiatr Serv. 2010 Jan 1;61(1):81–5.

50. Jones LE, Turvey C, Carney-Doebbeling C. Inadequate follow-up care for depression and its impact on antidepressant treatment duration among veterans with and without diabetes mellitus in the Veterans Health Administration. Gen Hosp Psychiatry. 2006 Dec;28(6):465–74.

51. When Depression is the Diagnosis, What Happens to Patients and Are They Satisfied? [Internet]. AJMC. [cited 2020 Nov 10]. Available from: https://www.ajmc.com/view/feb03-46p131-140

52. Common Mental Health Disorders - PHE [Internet]. [cited 2020 Sep 28]. Available from: https://fingertips.phe.org.uk/profile-group/mental-health/profile/common-mental-disorders/data#page/0/gid/1938132720/pat/46/par/E39000026/ati/165/are/E38000056/iid/90593/age/168/sex/4/cid/4/tbm/1/page-options/ovw-tdo-0_car-do-0_eng-vo-0_eng-do-0_ovw-do-0

53. Vedavanam S, Steel N, Broadbent J, Maisey S, Howe A. Recorded quality of care for depression in general practice: an observational study. Br J Gen Pract. 2009 Feb 1;59(559):e32–7.

54. Jaffe DH, Rive B, Denee TR. The humanistic and economic burden of treatment-resistant depression in Europe: a cross-sectional study. BMC Psychiatry. 2019 Aug 7;19(1):247.

55. Grembowski DE, Martin D, Patrick DL, Diehr P, Katon W, Williams B, et al. Managed Care, Access to Mental Health Specialists, and Outcomes Among Primary Care Patients with Depressive Symptoms. J Gen Intern Med. 2002 Apr;17(4):258–69.

56. Piek E, van der Meer K, Penninx BW, Verhaak PF, Nolen WA. Referral of patients with depression to mental health care by Dutch general practitioners: an observational study. BMC Fam Pract. 2011 May 26;12:41.

57. Lora A, Monzani E. La qualità della cura dei disturbi mentali gravi in Lombardia [Internet]. ASLMN; 2009. Available from: https://www.aslmn.net/docs_file/3_Qualita_Cura_Distrubi_Mentali_Gravi_Lombardia.pdf

58. Kunzel HE, Ackl N, Hatzinger M, Held K, Holsboer-Trachsler E, Ising M, et al. Outcome in delusional depression comparing trimipramine monotherapy with a combination of amitriptyline and haloperidol--a double-blind multicenter trial. J Psychiatr Res. 2009 Apr;43(7):702–10.

**Supplement 3: Items in survey to experts to indicate putative recommendations for minimising treatment gaps**

Round 1 items

| **Gap** | **Item** | **Outcome** |
| --- | --- | --- |
| 1/2 | Anti-stigma campaigns to encourage individuals to recognise depressive illness and seek help | Reconsider |
| 1/2 | Information provided to people who might be depressed and their loved ones, to inform them how best to seek help and what to expect from primary care | Reconsider |
| 1/2 | Service provision - increase availability (e.g. number, flexibility in times/dates) of primary care appointments | Reconsider |
| 1/2 | More provision of electronic primary care appointments (e.g. video) to increase scope for access to care | Reconsider |
| 1/2 | Increase the availability of longer appointments to maximise likelihood of depression screening | Reconsider |
| 1/2 | Introduce mandatory depression screening programmes in primary care; GP's have to assess patients' mood | Reject |
| 1/2 | Improve screening tools that primary care practitioners could use to detect depression more quickly / easily (e.g. through computerised speech analysis or passive data) | Reconsider |
| 1/2 | Advertisement of self-management e-mental health tools to help people recognise their own depression* | Reconsider |
| 1/2 | Integrate self-management e-mental health tools, with healthcare practice, so that primary care practitioners could see a person's depression symptom scores before an initial appointment* | Reconsider |
| 2 | More specific guidance for primary care practitioners about the need/benefits to treat people early with moderate or severe depression | Reconsider |
| 2/3 | Implement 'shared-care' arrangements e.g. psychiatrists design criteria and forms for nursing staff to complete with patients to manage treatment / indicate suitability for specific treatments / organise prescriptions, to increase efficiency and resources to access treatment | Endorse |
| 2/3 | Enhance the training of, and support for, other health professionals (e.g. nurses, pharmacists) to provide them with greater prescribing responsibilities, to increase efficiency of treatment provision | Reject |
| 2/3 | Anti-stigma or improved-information campaigns to provide patients with evidence based findings about the effectiveness of antidepressants, to reduce mis-information | Endorse |
| 2/3 | Tools to equip primary care practitioners to provide better information to inform patients about the potential benefits and harms of medication and psychological therapies (e.g. evidence-based information in a leaflet, longer appointments to allow fuller explanations) | Reconsider |
| 2/3 | Computerised decision-support tools to help primary care practitioners determine which treatment(s) to prescribe to people with moderate to severe depression | Reconsider |
| 2/3 | Integration of prescribing-support tools with existing electronic health records to increase efficiency and accurate detection of e.g. contraindications to treatments | Endorse |
| 2/3 | Increase the availability of psychological therapy access | Endorse |
| 2/3 | Encourage patient preference to inform initial treatment with medication or psychological therapy options | Endorse |
| 4 | Optimise self-management tools for patients to help manage their condition and feed back to healthcare providers during treatment* | Reconsider |
| 4 | Automatic appointment scheduling (and reminders) at suitable intervals after new treatment initiation | Reconsider |
| 4 | Screen for risk factors that would indicate need for more frequent follow-up e.g. polypharmacy, risk for bipolar, risk of suicidality, history of low treatment adherence or side effects | Endorse |
| 4 | Training for primary care physicians on when to follow up (in line with stepped care monitoring guidelines) | Reconsider |
| 4 | Increased availability of appointments to remove barriers to follow-up care | Endorse |
| 4 | Further develop provision of electronic primary care appointments (e.g. video, app) to increase ongoing access to primary care practitioners | Reconsider |
| 4 | Primary care physicians to incorporate standardised, ongoing assessment of depressive symptoms, to better monitor response and indicate if treatment changes are required (measurement-based care) | Reconsider |
| 4 | Integrate e-mental health tools for patients to rate their ongoing symptoms and side effects, with results accessible to healthcare practitioners, to indicate need for enhanced/reduced follow-up frequency* | Endorse |
| 5 | Specialist program for 'mental health specialist general practitioners': primary care physicians could train to have psychiatric expertise to enable better treatment of depressed people who would not reach secondary care | Reconsider |
| 5 | Integrate psychiatrists into primary care services to reduce the procedures needed for patients who are not responding well to treatments to access psychiatric input | Reconsider |
| 5 | Early screening for patients at risk for needing specialist treatment earlier in the care pathways, e.g. previous treatment resistance or a bipolar disorder | Endorse |
| 5 | Enhanced training programmes into psychiatry, to increase provision in secondary care mental health | Reconsider |
| 5 | Equipping general physicians with increased knowledge of which patients should be referred into secondary care services, and at which stage of care | Reconsider |
| 5 | Implement standards within secondary care services, regarding the number/frequency of contacts that referred patients should receive | Reject |
| 5 | Implement systems to ensure better transition for people after discharge from secondary to primary care e.g. joint working between psychiatric and general physicians, or occasional follow-up contacts within secondary care after discharge. | Reconsider |
| 5 | Resource input to create a greater number of specialist mood disorders centres | Endorse |
| 5 | Enhance education programmes to train psychiatrists / practitioners within secondary care to achieve a specialism in mood disorders | Endorse |

Round 2 items

| **Gap** | **Item** | **Outcome** |
| --- | --- | --- |
| 1/2 | Anti-stigma campaigns to encourage individuals to recognise depressive illness and seek help  *NB responses for this item in the last round were split between countries; after public awareness campaigns some people feel that instead of helping these lead to 'compassion fatigue', others (with or without these campaigns) feel that they encourage help seeking.* | Reject |
| 1/2 | Information provided to people who might be depressed and their loved ones, to inform them how best to seek help and what to expect from primary care | Reconsider |
| 1/2 | Service provision - increase availability (e.g. number, flexibility in times/dates) of primary care appointments | Reconsider |
| 1/2 | More provision of electronic primary care appointments (e.g. video) to increase scope for access to care | Reject |
| 1/2 | Increase the availability of longer appointments to maximise likelihood of depression screening | Reconsider |
| *1/2* | *Specifically, for screening people who have a recurrent depression, flag for general practitioners on the notes of people who have a documented history of depression, who could be prioritised for depression screening in future appointments.* | *Reject* |
| 1/2 | Improve screening tools that primary care practitioners could use to detect depression more quickly / easily (e.g. through computerised speech analysis or passive data) | Reject |
| 1/2 | Advertisement of self-management e-mental health tools to help people recognise their own depression (*independent of general practitioner)* | Reject |
| 1/2 | Integrate self-management e-mental health tools, with healthcare practice, so that primary care practitioners could see a person's depression symptom scores before an initial appointment* | Reconsider |
| 2 | More specific guidance for primary care practitioners about the need/benefits to treat people early with moderate or severe depression | Reject |
| 2/3 | Tools to equip primary care practitioners to provide better information to inform patients about the potential benefits and harms of medication and psychological therapies (e.g. evidence-based information in a leaflet, longer appointments to allow fuller explanations) | Endorse |
| *2/3* | *Provide information to patients with more practical information (separate from above) about psychological therapies to inform patient preference for treatments (e.g. options of different types available, waiting list times, cost in places where therapy is not free).* | *Endorse* |
| 2/3 | Computerised decision-support tools to help primary care practitioners determine which treatment(s) to prescribe to people with moderate to severe depression | Reconsider |
| *2/3* | *Addition of mental health workers (e.g. nurses) in primary care - NB this is not specifically for prescribing responsibilities (as in last round) but for general support to GP's. This is also not intended to be in replacement of physician input* | *Reconsider* |
| *2/3* | *Greater access to psychological therapies that are not just CBT based (e.g. dynamic, interpersonal, counselling-based etc.)* | Reconsider |
| 4 | Optimise self-management tools for patients to help manage their condition and feed back to healthcare providers during treatment *NB previous comment suggesting that self-management tools could be enhanced by including guidance by health professionals to support self management. Please rate your agreement/disagreement with this in mind.** | Reconsider |
| 4 | Automatic appointment scheduling (and reminders) at suitable intervals after new treatment initiation  *NB some commented that this already happens - we suspect it does in some areas more than others so please rate how important its implementation is regardless of whether it is happening now or not.* | Endorse |
| 4 | Training for primary care physicians on when to follow up (in line with stepped care monitoring guidelines) | Reject |
| 4 | Further develop provision of electronic primary care appointments (e.g. video, app) to increase ongoing access to primary care practitioners | Reconsider |
| 4 | Primary care physicians to incorporate standardised, ongoing assessment of depressive symptoms, to better monitor response and indicate if treatment changes are required (measurement-based care) | Reconsider |
| 5 | Specialist program for 'mental health specialist general practitioners': primary care physicians could train to have psychiatric expertise to enable better treatment of depressed people who would….. | Reconsider |
| 5 | Integrate psychiatrists into primary care services to reduce the procedures needed for patients who are not responding well to treatments to access psychiatric input. NB in response to previous comment, it may be that people feel this is unlikely to happen but in some UK practices it is starting (e.g. for 0.5 day per week) and one of the intentions of this survey is to help lobby public policy to improve resource provision. | Endorse |
| 5 | Enhanced training programmes into psychiatry, to increase provision in secondary care mental health | Endorse |
| 5 | Equipping general physicians with increased knowledge of which patients should be referred into secondary care services, and at which stage of care | Reconsider |
| 5 | Implement systems to ensure better transition for people after discharge from secondary to primary care e.g. joint working between psychiatric and general physicians, or occasional follow-up contacts within secondary care after discharge. | Endorse |
| 5 | *Not only encouraging referrals to secondary care, but enabling a looser criteria for patient referrals to be ACCEPTED (a problem at present is people being referred but referrals refused).* | Endorse |
| 5 | *Enable individuals to access more intensive (whether specialist care or not) treatment, removing barriers in terms of external employment/education processes e.g. campaigns to remove stigma and facilitate time of work/studying in terms of HR employment processes* | Reconsider |
| 5 | *Process for tertiary services to establish a structured long-term / follow-up plan with each patient (incorporating any associated comorbidity, treatment/adherence/tolerability, social support and coping strategies, future planning, active involvement or provision for the patient’s family/friends).* | Reconsider |

Items in italic were added based on suggestions and comments from round 1.

Round 3 items

| **Gap** | **Item** | **Outcome** |
| --- | --- | --- |
| 1/2 | Information provided to people who might be depressed and their loved ones, to inform them how best to seek help and what to expect from primary care | Endorse |
| 1/2 | Service provision - increase availability (e.g. number, flexibility in times/dates) of primary care appointments | Endorse |
| 1/2 | Increase the availability of longer appointments to maximise likelihood of depression screening | Endorse |
| 1/2 | Integrate self-management e-mental health tools, with healthcare practice, so that primary care practitioners could see a person's depression symptom scores before an initial appointment* | Endorse |
| 2/3 | Computerised decision-support tools to help primary care practitioners determine which treatment(s) to prescribe to people with moderate to severe depression | Endorse |
| 2/3 | Addition of mental health workers (e.g. nurses) in primary care - NB this is not specifically for prescribing responsibilities (as in last round) but for general support to GP's. This is also not intended to be in replacement of physician input | Reconsider |
| 2/3 | Greater access to psychological therapies that are not just CBT based (e.g. dynamic, interpersonal, counselling-based, etc.) | Reconsider |
| 4 | Optimise self-management tools for patients to help manage their condition and feed back to healthcare providers during treatment NB previous comment suggesting that self-management tools could be enhanced by including guidance by health professionals to support self management. Please rate your agreement/disagreement with this in mind.* | Endorse |
| 4 | Further develop provision of electronic primary care appointments (e.g. video, app) to increase ongoing access to primary care practitioners | Reconsider |
| 4 | Primary care physicians to incorporate standardised, ongoing assessment of depressive symptoms, to better monitor response and indicate if treatment changes are required (measurement-based care) | Endorse |
| 5 | Specialist program for 'mental health specialist general practitioners': primary care physicians could train to have psychiatric expertise to enable better treatment of depressed people who would….. | Endorse |
| 5 | Equipping general physicians with increased knowledge of which patients should be referred into secondary care services, and at which stage of care | Endorse |
| 5 | Enable individuals to access more intensive (whether specialist care or not) treatment, removing barriers in terms of external employment/education processes e.g. campaigns to remove stigma and facilitate time of work/studying in terms of HR employment processes | Endorse |
| 5 | Process for tertiary services to establish a structured long-term / follow-up plan with each patient (incorporating any associated comorbidity, treatment/adherence/tolerability, social support and coping strategies, future planning, active involvement or provision for the patient’s family/friends). | Endorse |

* Note related to high-quality self-management (e)tools: these could be multifaceted, with potential for effectiveness across care pathways. They could be developed at a large scale (where people could check their symptoms for a number of conditions and be targeted towards depression-specific tools if rating high symptoms of depression), and/or at a smaller scale (for example, if integrated with health records and reason for an appointment made is noted as relating to mental health beforehand, access to symptom rating scales that a clinician could see at the start of an appointment.) For use before a treatment starts, such a tool may even include a function for a patient to make treatment-related requests, which could reduce a gatekeeper role of physicians. During treatment, as well as the uses noted in Table 6, e-tools could also integrate guided self-help or psychoeducation tools or reminders to take medication / attend clinics. As digital tools are becoming more important during the coronavirus pandemic, this may provide additional opportunity for their development and evaluation (of impact on care for depression).
